# The Sealing Effect Improvement Prediction of Flat Rubber Ring in Roller Bit Based on Yeoh_Revised Model

**DOI:** 10.3390/ma15165529

**Published:** 2022-08-11

**Authors:** Wei Zhou, Chengwen Wang, Peng Fan, Yuchun Kuang, Zongzheng Dong

**Affiliations:** 1 School of Petroleum Engineering, China University of Petroleum (East China), Qingdao 266580, China; 2 Mechatronic Engineering College, Southwest Petroleum University, Chengdu 610500, China

**Keywords:** FRR, Yeoh_revised, Mises stress, incompressible

## Abstract

In a roller bit, the flat rubber ring (FRR) often needs to apply a certain amount of compression to ensure that its rotation and static sealing surfaces can be stably sealed. For the predicted Mises stress, values smaller than the actual Mises stress due to soft single-axis compression (SAC) stress are predicted by the Yeoh (N = 3) model. To more reasonably predict stress under the static compression of the FRR in the roller bit, the sealing effect of the FRR based on the SAC contact stress and the calculated Mises stress was evaluated by the Yeoh_revised model. Based on the assumption that hydrogenated nitrile-butadiene rubber (HNBR) is isotropic and incompressible, first, we derived the fitting formulas for three types of constitutive models and the Jacobi matrix of the Yeoh_revised model and developed hyperelastic constitutive subroutines. Simultaneously, the accuracy of three models (Yeoh, Yeoh_revised and Ogden) was evaluated by the goodness of fit (R^2^) to data from three kinds of tensile experiment tests. The highest R^2^ is 0.9771 with the Yeoh_revised model, which merges the advantages of the other two fitting models and effectively improves the Yeoh model’s soft property of SAC contact stress. Additionally, by measuring on-site FRR wear, the maximum Mises stress on the sealing surface calculated based on the Yeoh_revised model is about twice that of the Yeoh model, and the maximum Mises stress on the rotation contact sealing surface is higher than that on the outside (static sealing) surface, which makes the aging of the rotation surface more severe. Thus, it was demonstrated that, on the premise of ensuring FRR sealing contact stress, the Yeoh_revised model can more reasonably predict the sealing effect of the FRR to more precisely calculate Mises stress than the Yeoh model. This also contributes to FRR structure optimization to prolong the service life of the FRR in the roller bit.

## 1. Introduction

Generally speaking, the rubber of the FRR used in a roller bit is an initially isotropic and incompressible material. The study of its mechanical constitutive model is mainly divided into two categories: phenomenological theory and thermodynamic statistical theory based on molecular chain networks. Based on the former theory, Mooney, M. [1] and Rivlin, R. S. [2] proposed the classic Mooney–Rivlin (M-R) hyperelastic constitutive model, which represents the strain energy density function with deformation tensor invariant I_i_ (i = 1, 2, 3). The second-order term of this model is the most widely used hyperelastic constitutive model in current small deformation analyses, with significant errors based on the Neo-Hookean model when fitting experimental data with a tensile ratio greater than 1.4. On the basis of the Mooney–Rivlin model, O.H. Yeoh [3] only considered the effect on the strain energy density function from the first strain invariant (I_1_) while retaining (I_1_-3) at less than or equal to the third-order terms to accurately predict the uniaxial and plane tensile test stress–strain relationship, but the “soft” phenomenon was observed compared to experimental data. Ogden, R. W. [4] represented the strain energy density function with the elongation ratio λ_i_ (i = 1, 2, 3), which can accurately predict the stress–strain data of uniaxial stretching, plane stretching and equibiaxial stretching experiments simultaneously, but it is not suitable for predicting single-stretching experimental data.

Based on the latter theory, Guth et al. [5] proposed the Neo-Hookean model, called the classical Gaussian statistical mechanics model, which is only suitable for small deformation situations. Lopez-Pamies, O. [6] proposed an evolving porous Neo-Hookean model to characterize the macroscopic response of 2D isotropic porous Neo-Hookean solids with random and particulate microstructures, providing ample motivation to carry out further analyses for more general 3D material systems. Treloar, L. R. G., et al. [7] and Treloar, L. R. G. and G. Riding [8] proposed that the Neo-Hookean model no longer applies when the end of a Gaussian chain exceeds the vector length by 1/3 of the chain length. Guth, E. et al. [5] and Wang, M. C. and E. Guth [9] modified the ideal Gaussian theory under the distribution of the Langevin inverse function, which more accurately simulates the nonlinear elastic characteristics of rubber materials under large deformation, and further proposed a simplified three-chain model. ARRUDA, E. M. and M. C. Boyce [10] proposed an eight-chain molecular model based on the three-chain non-Gaussian network model and showed that the Arruda–Boyce model may be preferable when only uniaxial tensile test data are available. On the previous basis, P. D. Wu and E. van der Giessen [11] established a more accurate full network model, which can fit between the three-chain and eight-chain molecular models. Yang, L. and L. Yang [12] proposed that under certain assumptions, the Gent model [13,14] and the Arruda–Boyce model are consistent. Lopez-Pamies, O. [15] proposed a two-term model, which can be regarded as a generalized Neo-Hookean model, as well as a variant of the Arruda–Boyce (eight-chain) model, and can degenerate into the Yeoh configuration, so it also fits equibiaxial stretching experimental data with the soft phenomenon. Based on this model, Huang, Z.-P. [16] proposed a Gent-Gent hyperelastic constitutive model, which improved the Gent fit. Zhou, L. et al. [17] provided further evidence of the good performance of the equibiaxial deformation prediction of the Gent-Gent model by using it to study the inflation of a circular plane membrane.

Based on the experimental data of L. R. G. TRELOAR [18,19], Xiao-ling, H. et al. [20] fitted five common prediction models for stress–strain test data (Neo-Hookean, Mooney–Rivlin, Yeoh, Ogden and Arruda–Boyce) and employed a model selection strategy for three basic test datasets. The results show that only Ogden (N = 3) could completely fit the experimental data when the three types of experiments were sufficient, avoiding the soft phenomenon of equibiaxial tensile stress fitting. Xue-bing, L. and W. Yin-tao [21] proposed a revised Yeoh hyperelastic material constitutive model that effectively overcomes the “partial soft” properties of the Yeoh model in predicting equibiaxial stretching curves. In a large strain range, the stress–strain relationship of uniaxial, plane and equibiaxial tension compression could be accurately predicted simultaneously, but it was not compared with the Ogden (N = 3) model and needs to be verified by the finite element method (FEM).

Among the constitutive models used in FRR seal analysis, the M-R model is the most extensively used; for instance, Niu, S [22] and Zhang, J. and J. Xie. [23] investigated the sealing performance of an O-ring based on FEM with the M-R model, where the effects of pressure and pre-compression, fluid pressure, the friction coefficient, etc., on the sealing performance analysis were studied. In addition, Liao, B. et al. [24] obtained the time-varying reliability of the O-ring, and Hu, Y. et al. [25] studied the fretting wear of the O-ring, as well as the life prediction, safety and service life of the seal. Yi Zhou and L. Wang [26] proposed that O-ring rubber circles are superior to other cross-sectional rubber rings, while Zhang, H. and J. Zhang [27] reported that the O-ring can be effectively replaced by a D-ring in a static seal. Liang, B. et al. [28] proposed that the material and geometric parameters had a greater influence on the reliability of the rubber O-ring, so Zhang, L. and X. Wei. [29] proposed a new structure of a butterfly rubber ring by analyzing the factors affecting the sealing performance of the rubber ring on the groove side. The Yeoh model is also widely used. Zhou, C. et al. [30,31] studied the sealing performance of a combined sealing structure composed of a rubber D-ring, O-ring and wedge ring used in a high-pressure hydrogen storage vessel by elucidating the swelling mechanisms due to dissolved hydrogen. 

The Yeoh_revised constitutive model is the most accurate in fitting experimental data (R^2^ = 0.9771) compared to Yeoh and Ogden models and is especially suitable for predicting Mises and contact stresses under SAC deformation. In this study, we evaluated the sealing effect of SAC contact stress and Mises stress based on the Yeoh_revised model for a novel FRR in a roller bit under SAC deformation. The results clearly show that the Yeoh_revised model can predict the Mises stress more accurately than the Yeoh model and provides a more reasonable prediction of the aging of the FRR, which further ensures a more stable seal. It also provides specifications for its size optimization in the future.

## 2. Problem Description and Analysis Method

### 2.1. FRR Static Compression Analysis in a Roller Bit

FRRs are often used for sealing in roller bits. The sealing effect is related to the influence of the working environment, such as the moving force in the roller bit, down-hole temperature, ambient pressure, etc. [32,33]. During the sealing process, the contact stress of the sealing surface is related to the amount of FRR compression, the fluid pressure difference on both sides of the FRR, and FRR hardness. Generally speaking, higher ambient temperature and internal Mises stress tend to accelerate the aging of rubber [24,25], resulting in rubber hardness increases that make the wear of the sealing surface inconsistent [34]. The higher the hardness of the FRR surface, the slower the wear. Due to inconsistent hardness, a wear gradient of the FRR in the roller bit will occur, and the sealing effect of the FRR will finally deteriorate. 

To reduce the difference in wear, this study calculated the contact stress and Mises stress of an FRR in the static sealing process based on the Yeoh_revised model and compared it to the calculation results of the Yeoh model. The aim is to achieve a more accurate prediction of the maximum Mises stress on the FRR under the premise of ensuring a stable seal. Meanwhile, it provides a reference for reasonably designing the size of the FRR structure, prolonging the service life and ensuring a more reliable seal. 

**Figure 1:**
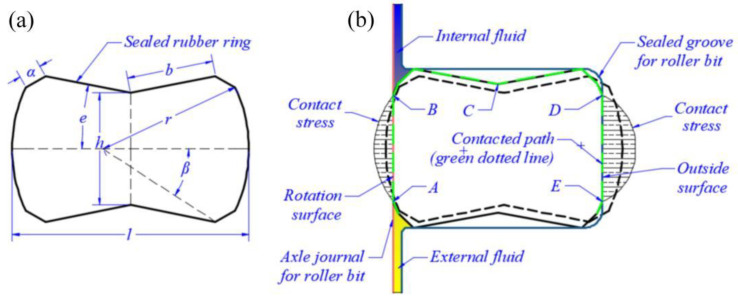
Schematic diagram of the cross-sectional structure (**a**) and contact deformation of FRR (**b**) in a roller bit. In the contact path (green dotted line in Figure 1b), the critical point on the rotation surface contact with the outer and inner fluid zone is A and B, point C is the middle point in the contact path and the critical point on the outside surface contact with the outer and inner fluid zone is D and E.

The novel structure scheme of the FRR is shown in Figure 1a, in which the center symmetry form and structural parameters are as follows: seal length *l* = 6.25 mm, height *h* = 3.0 mm, oblique length *b* = 2.3 mm, arc radius *r* = 3.84 mm, oblique horizontal angle *e* = 11°, arc corresponding to central angle *β* = 25°, oblique chamfer length *α* = 0.6 mm, etc.

Figure 1b is the schematic diagram of the FRR force in the roller drill, where the outside surface and rotation surface of the FRR are pre-compressed by a sealed groove and axle journal, respectively. During the actual sealing process, the two ends of the rotation surface profile of the FRR are subjected to fluid pressure (e.g., 20 MPa). Meanwhile, to simplify the calculation, the ambient temperature is assumed to be 120 °C, the friction factor of the sealing contact surface is 0.08, the sealed groove and axle journal are rigid bodies only considering FRR deformation, and the contact, load and constraint of the whole model have axial symmetry. Cloud diagrams of FEM calculation results for contact stress and Mises stress under FRR static compression are shown in Section 3.2. By setting the contact path (Figure 1b), the contact stress and Mises stress curves can be obtained as shown in Section 3.2 too.

### 2.2. Hyperelastic Experiment and Fitted Model 

**Figure 2:**
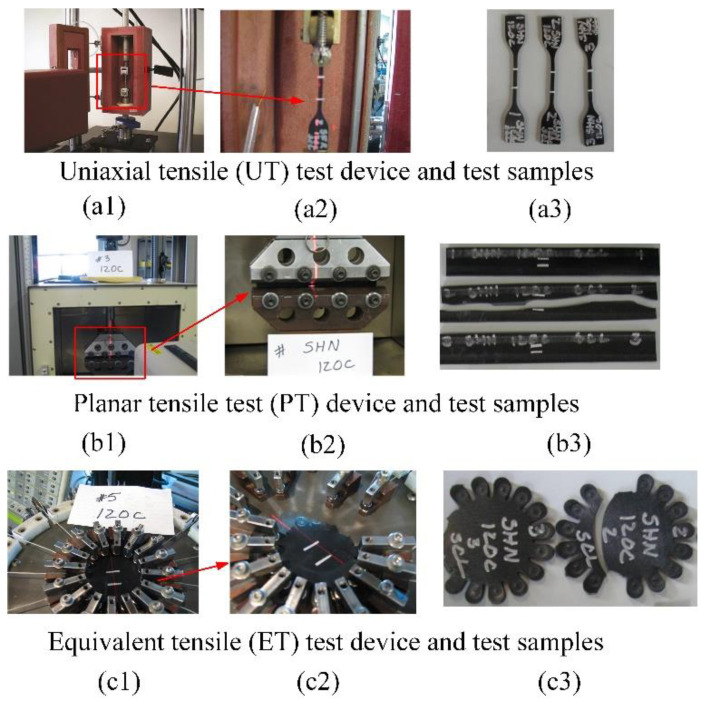
Three tensile test devices (**a1**–**c1**), test section magnification (**a2**–**c2**) and test samples’ extrinsic features (**a3**–**c3**) after tests.

**Figure 3:**
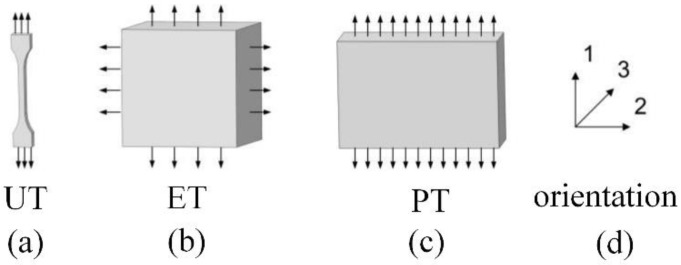
Schematic representation of uniaxial tensile (**a**), equivalent tensile (**b**), planar tensile (**c**) of stretched samples and deformation directions (**d**).

To determine the hyperelastic constitutive model of HNBR materials, uniaxial tensile (UT), planar tensile (PT) and equivalent tensile (ET) tests [35,36,37] at 120 °C were performed based on the good resistance of HNBR at high temperatures, as shown in Figure 2. In the basic dense elastomer hyperelastic tensile experiment executed by Axel Products, Inc., the parameters were set as follows: strain speed: 0.01 s^−1^, five times per level; strain levels: 11%, 24%, 30% and 39%. Three UT specimens, three PT specimens and three ET specimens were cut from the provided slabs (slab measurements: 150 mm in length by 150 mm in width, 1.0 to 2.0 mm thick). In the test, the specimens were loaded slowly with between zero force and a user-defined stretch level for 5 loadings and unloadings at up to 4 maximum strain levels so as to examine the initial stress–strain behavior and the “stabilized” stress–strain behavior in each of the maximum strain conditions. In addition, a schematic diagram of the tensile sample force is given in Figure 3. Considering the HNBR as an isotropic incompressible material, the main tensile ratio *λ*_i_ (i = 1,2,3) and the Cauchy stress in related directions (Figure 3), Equation (1) is obtained.

(1) $$\left\{\begin{array}{l} ST:\lambda_{1}=\lambda ,\lambda_{2}=\lambda^{-0.5},\lambda_{3}=\lambda^{-0.5},\sigma_{2}=\sigma_{3}=0\\ ET:\lambda_{1}=\lambda ,\lambda_{2}=\lambda ,\lambda_{3}=\lambda^{-2},\sigma_{3}=0\\ PT:\lambda_{1}=\lambda ,\lambda_{2}=1,\lambda_{3}=\lambda^{-1},\sigma_{3}=0 \end{array}\right.$$

#### 2.2.1. Mathematical Stress Formula 

HNBR materials are usually regarded as incompressible and isotropic hyper-elastomers, and their mechanical properties can be described by the strain energy density equation. Under isothermal conditions, the strain energy density equation of isotropic super-elastic materials can be represented as in Equation (2) as a function of the three strain invariants of the left/right Cauchy–Green deformation tensor ***b***/***C***, as defined in Equation (3).

(2) $$\phi =\phi (I_{C},II_{C},III_{C}),\phi =\phi (I_{b},II_{b},III_{b})$$

in which:

(3) $$\left\{\begin{array}{l} I_{b}=I_{C}=trC=\lambda_{1}{}^{2}+\lambda_{2}{}^{2}+\lambda_{3}{}^{2}\\ II_{b}=II_{C}=\frac{1}{2}[{\left(trC\right)}^{2}-trC^{2}]=\lambda_{1}{}^{2}\lambda_{2}{}^{2}+\lambda_{1}{}^{2}\lambda_{3}{}^{2}+\lambda_{2}{}^{2}\lambda_{3}{}^{2}\\ III_{b}=III_{C}=\det C=\lambda_{1}{}^{2}\lambda_{2}{}^{2}\lambda_{3}{}^{2} \end{array}\right.$$

By definition, the relationship between type II Piola–Kirchhoff stress ***S*** and strain energy density *ϕ* (strain energy per unit volume) is expressed as Equation (4) [13].

(4) $$S=2\frac{\partial \phi }{\partial C}$$

Combining Equation (2) with Equation (3) and deriving Equation (4) by the chain rule of the composite function [21], we derived Equation (5).

(5) $$S=2\frac{\partial \phi }{\partial C}=2[(\frac{\partial \phi }{\partial I_{C}}+I_{C}\frac{\partial \phi }{\partial II_{C}})I-\frac{\partial \phi }{\partial II_{C}}C+III_{C}\frac{\partial \phi }{\partial III_{C}}C^{-1}]$$

We consider the relationship between type II Piola–Kirchhoff stress ***S*** and Cauchy stress ***σ*** as in Equation (6) and combine $C=F^{T}\cdot F,b=F\cdot F^{T},I_{b}=I_{C},II_{b}=II_{C},III_{b}=III_{C}$ and $J={\left(III_{b}\right)}^{0.5}$. Using the Cayley–Hamilton theorem [38] in Equation (7), Equation (6) can be transformed into Equation (8).

(6) $$\sigma =\frac{1}{J}F\cdot S\cdot F^{T}$$

(7) $$-b^{2}=-I_{b}b+II_{b}I-III_{b}b^{-1}$$

(8) $$\begin{array}{rr} \sigma & =\frac{2}{{\left(III_{b}\right)}^{\frac{1}{2}}}[(\frac{\partial \phi }{\partial I_{b}}+{Ι}_{C}\frac{\partial \phi }{\partial II_{b}})b-\frac{\partial \phi }{\partial II_{b}}b^{2}+III_{b}\frac{\partial \phi }{\partial III_{b}}I]\\ & =\frac{2}{{\left(III_{b}\right)}^{\frac{1}{2}}}[\frac{\partial \phi }{\partial I_{b}}b-\frac{\partial \phi }{\partial II_{b}}III_{b}b^{-1}+(II_{b}\frac{\partial \phi }{\partial II_{b}}+III_{b}\frac{\partial \phi }{\partial III_{b}})I]\\ & =-PI+\frac{\partial \phi }{\partial I_{b}}b-\frac{\partial \phi }{\partial II_{b}}b^{-1} \end{array}$$

Depending on the incompressibility of the rubber material, $ΙΙ{Ι}_{b}=ΙΙ{Ι}_{C}=1$, and ***P*** is hydrostatic pressure. For the isotropic materials, ***σ***, ***b*** and ***b***^−1^ are coaxial [39]; therefore, in the spindle coordinate system, the principal Cauchy stress in Equation (9a) and the nominal principal stress in Equation (9b) are obtained.

(9a) $$\sigma_{i}=-P+2\frac{\partial \phi }{\partial I_{b}}\lambda_{i}{}^{2}-2\frac{\partial \phi }{\partial II_{b}}\lambda_{i}{}^{-2}$$

(9b) $$P_{i}=\frac{\sigma_{i}}{\lambda_{i}}=\frac{-P}{\lambda_{i}}+2\frac{\partial \phi }{\partial I_{b}}\lambda_{i}-2\frac{\partial \phi }{\partial II_{b}}\lambda_{i}{}^{-3}$$

Eliminating uncertain hydrostatic pressure terms, Equation (10) is as follows.

(10) $$\sigma_{i}-\sigma_{j}=2\frac{\partial \phi }{\partial I_{b}}(\lambda_{i}{}^{2}-\lambda_{j}{}^{2})-2\frac{\partial \phi }{\partial II_{b}}(\lambda_{i}{}^{-2}-\lambda_{j}{}^{-2})$$

#### 2.2.2. The Three Models’ (Yeoh (N = 3), Yeoh_Revised (N = 3) and Ogden (N = 3)) Fitted Stress

Yeoh (N = 3) uses the higher-order term of I_1_ to correct the strain energy function of the Neo-Hookean model. The modified model is called the Reduced Polynomial model, whose strain energy function is expressed in Equation (11). Based on Drucker stability [40], the material constraint inequalities are defined in Equation (12).

(11) $$\phi =C_{10}(I_{1}-3)+C_{20}{\left(I_{1}-3\right)}^{2}+C_{30}{\left(I_{1}-3\right)}^{3}$$

(12) $$C_{10}>0,C_{20}<0,C_{30}>0$$

Combining Equations (9b) and (10), the relationship between the nominal stress ***P_i_*** and the stretching ratio *λ_i_* of the Yeoh (N = 3) model under three basic deformation modes is obtained, as shown in Equation (13) [38].

(13) $$\left\{\begin{array}{l} P_{yeoh}^{ST}=\sum_{i=1}^{3}2iC_{i0}{\left(\lambda^{2}+2\lambda^{-1}-3\right)}^{i-1}(\lambda -\lambda^{-2})\\ P_{yeoh}^{ET}=\sum_{i=1}^{3}2iC_{i0}{\left(2\lambda^{2}+\lambda^{-4}-3\right)}^{i-1}(\lambda -\lambda^{-5})\\ P_{yeoh}^{PT}=\sum_{i=1}^{3}2iC_{i0}{\left(\lambda^{2}+\lambda^{-2}-2\right)}^{i-1}(\lambda -\lambda^{-3}) \end{array}\right.$$

Since the *II_C_* term in Equation (2) is completely discarded, the Yeoh model predicts the ET stress with the “soft” phenomenon. Based on the above considerations, by introducing the first part of *II_C_* [20], the following strain energy equation is proposed (see Equation (14)), and based on Drucker stability [40], the material constraint inequality is defined in Equation (15).

(14) $$\phi =C_{10}(I_{1}-3)+C_{20}{\left(I_{1}-3\right)}^{2}+C_{30}{\left(I_{1}-3\right)}^{3}+C_{01}(I_{2}-3)$$

(15) $$C_{10}>0,C_{20}<0,C_{30}>0,C_{01}>0$$

Combining Equations (9b) and (10), the relationship between the nominal stress *P_i_* and the stretching ratio *λ_i_* of the Yeoh_revised (N = 3) model under three basic deformation modes is obtained, as shown in Equation (16).

(16) $$\left\{\begin{array}{l} P_{yeoh\_re}^{ST}=\sum_{i=1}^{3}2iC_{i0}{\left(\lambda^{2}+2\lambda^{-1}-3\right)}^{i-1}(\lambda -\lambda^{-2})+2C_{01}(1-\lambda^{-3})\\ P_{yeoh\_re}^{ET}=\sum_{i=1}^{3}2iC_{i0}{\left(2\lambda^{2}+\lambda^{-4}-3\right)}^{i-1}(\lambda -\lambda^{-5})+2C_{01}(\lambda^{3}-\lambda^{-3})\\ P_{yeoh\_re}^{PT}=\sum_{i=1}^{3}2iC_{i0}{\left(\lambda^{2}+\lambda^{-2}-2\right)}^{i-1}(\lambda -\lambda^{-3})+2C_{01}(\lambda -\lambda^{-3}) \end{array}\right.$$

The strain energy function of the Ogden model is shown in Equation (17) [4,38], where *μ_i_* and *α_i_* are arbitrary constants (which can be non-integers). Based on Drucker stability [40], material constraint inequalities are shown in Equation (18).

(17) $$\phi =\sum_{i=1}^{N}\frac{\mu_{i}}{\alpha_{i}}(\lambda_{1}{}^{\alpha_{i}}+\lambda_{1}{}^{\alpha_{i}}+\lambda_{1}{}^{\alpha_{i}}-3)$$

(18) $$\overset{N}{\underset{i=1}{\sum }}\mu_{i}\alpha_{i}>0$$

Combining Equations (9b) and (10), the relationship between the nominal stress *P_i_* and the stretching ratio *λ_i_* of the Ogden (N = 3) model under three basic deformation modes is obtained as shown in Equation (19).

(19) $$\left\{\begin{array}{l} P_{ogden}^{ST}=2\sum_{i=1}^{3}\frac{\mu_{i}}{\alpha_{i}}(\lambda^{\alpha_{i}-1}-\lambda^{-\frac{1}{2}\alpha_{i}-1})\\ P_{ogden}^{ET}=2\sum_{i=1}^{3}\frac{\mu_{i}}{\alpha_{i}}(\lambda^{\alpha_{i}-1}-\lambda^{-2\alpha_{i}-1})\\ P_{ogden}^{PT}=2\sum_{i=1}^{3}\frac{\mu_{i}}{\alpha_{i}}(\lambda^{\alpha_{i}-1}-\lambda^{-\alpha_{i}-1}) \end{array}\right.$$

#### 2.2.3. Model Goodness of Fit

Using the hyperelastic constitutive fitted model obtained in Section 2.2.2, least squares fitting was performed on experimental data [18,32]. The model goodness of fit *R*^2^ was introduced to evaluate the fitting quality, and the deviation square sum (SSdev) and the residual square sum (SSres) were calculated.

(20a) $$R^{2}=1-\frac{SS_{res}}{SS_{dev}}$$

(20b) $$SS_{res}={\sum_{i=1}^{N}\left({\overset{\wedge }{P}}_{i}-P_{i}\right)}^{2}$$

(20c) $$SS_{dev}={\sum_{i=1}^{N}\left(P_{i}-{\bar{P}}_{i}\right)}^{2}$$

where *P_i_* is the experimental value; $\bar{P_{i}}$ is the average of *P_i_*; $\hat{P_{i}}$ is the model fit value; and N is the number of test data points involved in fitting. A larger goodness-of-fit value, *R*^2^, indicates the higher overall goodness of the model fit. The Yeoh fitting formula in Equation (13), Yeoh_revised fitting formula in Equation (16) and Ogden fitting formula in Equation (19) were fitted to three sets of tensile experimental data. The fitting results and residual analysis are shown in Figures 6 and 7, respectively.

### 2.3. Validation of the Fitted Constitutive Parameters in FEM 

In engineering, the vast majority of dense HNBR materials are often considered mechanically incompressible during the deformation process [41,42], which still satisfies the accuracy requirements of the solution. In general, the hyperelastic Jacobi matrix of incompressible HNBR materials is often solved in the form of the stress–strain rate; $\overset{•}{S},\overset{•}{\tau }$ is not objective [39], so $C^{\tan}$ cannot be used for the hyperelastic Jacobi matrix of rubber materials [39]. Since $\overset{o}{\tau }$ and $\overset{□}{\tau }$ are objective, the Jacobi matrix that can be used to solve the constitutive model according to its objectivity is shown in Equations (21a) and (21b). Finally, the Jacobi matrix ***C_ijkl_*** of the material is derived from the variation in the Jaumann-Zaremba rate of the Kirchhoff stress tensor [39].

(21a) $$\left\{\begin{array}{l} \overset{•}{S}=C^{tan}:\overset{•}{E}\\ \overset{□}{\tau }=\overset{•}{\tau }-l\cdot \tau -\tau \cdot l^{T}\\ F^{-1}\cdot \overset{□}{\tau }\cdot F^{-T}=C^{tan}:F^{T}\cdot D\cdot F\\ \overset{□}{\tau }=(F\bar{⊗}F:C^{tan}:F^{T}\underline{⊗}F^{T}):D=L:D \end{array}\right.$$

(21b) $$\left\{\begin{array}{l} \overset{\circ }{\tau }=\overset{□}{\tau }+D\cdot \tau +\tau \cdot D=\overset{\wedge }{L}:D\\ D\cdot \tau +\tau \cdot D=2H:D\\ \overset{\wedge }{L}=L+2H\\ C_{ijkl}={\hat{L}}_{ijkl}/J=(L_{ijkl}+2H_{i}{}_{jkl})/J \end{array}\right.$$

where $C^{\tan}$ is known as the material tangent elasticity tensor, $\overset{•}{\tau }$ is the derivative of the Kirchhoff stress tensor with time, $\overset{□}{\tau }$ is the Oldroyd rate of the Kirchhoff stress tensor, $\overset{o}{\tau }$ is the Jaumann-Zaremba rate of the Kirchhoff stress tensor, L or $\hat{L}$ is the spatial tangent elasticity tensor in the current configuration, **H** is the symmetric part of $\hat{L}$, ***l*** is the velocity deformation gradient tensor, and **D** is the symmetric part of ***l***.

#### 2.3.1. Calculation of Yeoh_Revised Jacobi Matrix (Incompressible) 

In order to verify the correctness of the HNBR hyperelastic configuration model, this section first establishes the Jacobi matrix of the Neo-Hookean model and compares the results with the Neo-Hookean Jacobi matrix given by the help document in ABAQUS [43]. Helmholtz free energy per unit reference volume (ϕ) of the Neo-Hookean model is shown in Equation (22) [44,45].

(22) $$\phi =\tilde{\phi }+\phi^{vol}=C_{10}(I_{\tilde{C}}-3)+\frac{1}{D_{1}}{\left(J-1\right)}^{2}$$

where $\tilde{\phi },\phi^{vol}$ represent the shear and dilatation parts of Helmholtz free energy, respectively, and *D_i_* characterizes the compressibility of HNBR. 

**Figure 4:**
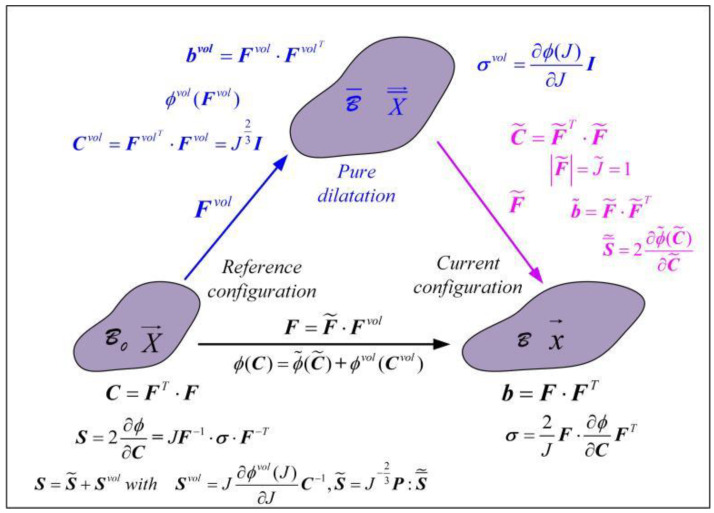
Multiplicative decomposition of deformation.

According to the definitions in Equations (4) and (5), the second type of Piola–Kirchhoff stress (***S***) is shown in Equation (23a), where $\tilde{S},S^{vol}$ represent the shear and dilatation parts of Piola–Kirchhoff stress (***S***), $\tilde{\bar{S}}$ represents the stress (***S***) defined in the pure dilatation configuration (Figure 4), and ***P*** is the fourth-order projection tensor.

(23a) $$S=2\frac{\phi (C)}{\partial C}=\tilde{S}+S^{vol}$$

(23b) $$\begin{array}{l} S^{vol}=J\frac{\phi^{vol}(J)}{\partial J}C^{-1}=\frac{2}{D_{1}}J(J-1)C^{-1}\\ \tilde{S}=2\frac{\partial \tilde{\phi }(\tilde{C})}{\partial C}=J^{-\frac{2}{3}}[\frac{\partial \tilde{C}}{\partial C}:2\frac{\partial \tilde{\phi }(\tilde{C})}{\partial \tilde{C}}]=J^{-\frac{2}{3}}P:\tilde{\bar{S}}=J^{-\frac{2}{3}}{\tilde{\bar{S}}}^{dev} \end{array}$$

(23c) $$\tilde{\bar{S}}=2\frac{\partial \tilde{\phi }(\tilde{C})}{\partial (\tilde{C})},\;P=\frac{\partial \tilde{C}}{\partial C}=II-\frac{1}{3}C^{-1}⊗C$$

Substituting Equations (22), (23b) and (23c) into Equation (23a), the specific expression of S is obtained in Equation (24).

(24) $$S=\tilde{S}+S^{vol}=2C_{10}J^{-\frac{2}{3}}[I-\frac{trC}{3}C^{-1}]+\frac{2}{D_{1}}J(J-1)C^{-1}$$

To determine the Neo-Hookean Jacobi matrix, Equation (24) is substituted into Equation (21a) to obtain the material tangent elasticity tensor ($C^{\tan}$), as shown in Equation (25), and the spatial tangent elasticity tensor (L) is shown in Equation (26).

(25) $$\begin{array}{l} C^{\tan}=2\frac{\partial S}{\partial C}=-\frac{4}{3}C_{10}J^{-\frac{2}{3}}[C^{-1}⊗I+I⊗C^{-1}]\\ +[\frac{4}{9}C_{10}J^{-\frac{2}{3}}trC+\frac{2}{D_{1}}(2J-1)J]C^{-1}⊗C^{-1}\\ +[\frac{2}{3}C_{10}J^{-\frac{2}{3}}trC-\frac{2}{D_{1}}(J^{2}-J)][C^{-1}\bar{⊗}C^{-1}+C^{-1}\underline{⊗}C^{-1}] \end{array}$$

(26) $$\begin{array}{l} L=F\bar{⊗}F:C^{\tan}:F^{T}\underline{⊗}F^{T}=-\frac{4}{3}C_{10}J^{-\frac{2}{3}}[I⊗b+b⊗I]\\ +[\frac{4}{9}C_{10}trbJ^{-\frac{2}{3}}+\frac{2}{D_{1}}(2J-1)J]I⊗I\\ +[\frac{2}{3}C_{10}trbJ^{-\frac{2}{3}}-\frac{2}{D_{1}}(J^{2}-J)][I\bar{⊗}I+I\underline{⊗}I] \end{array}$$

In fact, the material Jacobi matrix is obtained from the variation in the Jaumann-Zaremba rate of the Kirchhoff stress tensor ($\overset{o}{\tau }$). According to the relationship between $\overset{o}{\tau }$ and $\overset{□}{\tau }$ (see Equation (21b)), ***H_ijkl_*** and ${\hat{L}}_{ijkl}$ component expressions are shown in Equations (27) and (28), respectively. The detailed derivation process of ***H_ijkl_*** is shown in Appendix A.

(27) $$\begin{array}{l} 2H_{ijk}{}_{l}=C_{10}(\delta_{i}{}_{k}{\tilde{b}}_{jl}+\delta_{i}{}_{l}{\tilde{b}}_{jk}+{\tilde{b}}_{i}{}_{k}\delta_{jl}+{\tilde{b}}_{i}{}_{l}\delta_{jk})\\ -[\frac{2}{3}C_{10}\frac{tr\tilde{b}}{3}-\frac{2}{D_{1}}(J^{2}-J)](\delta_{i}{}_{k}\delta_{jl}+\delta_{il}\delta_{jk}) \end{array}$$

(28) $$\begin{array}{l} {\overset{\wedge }{L}}_{ijkl}=L_{ijkl}+2H_{ijkl}=-\frac{4}{3}C_{10}[\delta_{ij}{\tilde{b}}_{kl}+{\tilde{b}}_{ij}\delta_{kl}]\\ +[\frac{4}{9}C_{10}tr\tilde{b}+\frac{2}{D_{1}}(2J-1)J]\delta_{ij}\delta_{kl}\\ +C_{10}(\delta_{ik}{\tilde{b}}_{jl}+\delta_{il}{\tilde{b}}_{jk}+{\tilde{b}}_{ik}\delta_{jl}+{\tilde{b}}_{il}\delta_{jk}) \end{array}$$

Finally, the Neo-Hookean Jacobi matrix component ***C_ijkl_*** of rubber material is shown in Equation (29). The result is consistent with the help document given in the ABAQUS help file [46].

(29) $$\begin{array}{l} C_{ijkl}={\overset{\wedge }{L}}_{ijkl}/J=-\frac{4}{3J}C_{10}[\delta_{ij}{\tilde{b}}_{kl}+{\tilde{b}}_{ij}\delta_{kl}]\\ +[\frac{4}{9J}C_{10}tr\tilde{b}+\frac{2}{D_{1}}(2J-1)]\delta_{ij}\delta_{kl}\\ +\frac{C_{10}}{J}(\delta_{ik}{\tilde{b}}_{jl}+\delta_{il}{\tilde{b}}_{jk}+{\tilde{b}}_{ik}\delta_{jl}+{\tilde{b}}_{il}\delta_{jk}) \end{array}$$

Likewise, based on Equation (14), the strain energy density equation for the Yeoh_revised (incompressible) model is obtained [38] (see Equation (30)).

(30) $$\phi =\tilde{\phi }+\phi^{vol}=\sum_{i=1}^{3}C_{i0}{\left(I_{C}-3\right)}^{i}+C_{01}(II_{C}-3)+\gamma (J-1)$$

where $\tilde{C}=C$, and γ is the introduced Lagrange multiplier and satisfies $\gamma =-P=\sigma_{kk}/3$.

Equation (30) is substituted into Equation (23a), and after the calculation of Equation (31), Equation (32) is obtained.

(31) $$\begin{array}{l} S^{vol}=J\frac{\phi^{vol}(J)}{\partial J}C^{-1}=J\gamma C^{-1}\\ \tilde{S}=2\frac{\partial \tilde{\phi }(\tilde{C})}{\partial C}=2\left[(\frac{\partial \tilde{\phi }(\tilde{C})}{\partial I_{C}}+\frac{\partial \tilde{\phi }(\tilde{C})}{\partial II_{C}}I_{C})I-\frac{\partial \tilde{\phi }(\tilde{C})}{\partial II_{C}}C\right]\\ =\sum_{i=1}^{3}2iC_{i0}(I_{C}-3)i-1I+2C_{01}(I_{C}I-C) \end{array}$$

(32) $$S=\tilde{S}+S^{vol}=\sum_{i=1}^{3}2iC_{i0}{\left(I_{C}-3\right)}^{i-1}I+2C_{01}(I_{C}I-C)+J\gamma C^{-1}$$

Similar to the calculation of Equations (25)–(28), the Yeoh_revised model (incompressible) Jacobi matrix component ***C_ijkl_*** of HNBR material is shown in Equation (33), and the detailed derivation process is shown in Appendix B.

(33) $$\begin{array}{l} C_{ijkl}={\overset{\wedge }{L}}_{ijkl}/J=\frac{1}{J}[8C_{20}+24C_{30}trb-72C_{30}-4C_{01}]b_{ij}b_{kl}\\ -\frac{4C_{01}}{J}b_{ik}b_{jl}+\frac{1}{J}[J\gamma ]\delta_{ij}\delta_{kl}+\frac{1}{J}[C_{10}+2C_{20}(trb-3)\\ +3C_{30}(trb-3)2+C_{01}trb](\delta_{ik}b_{jl}+\delta_{il}b_{jk}+b_{ik}\delta_{jl}+b_{il}\delta_{jk})\\ -\frac{1}{J}C_{01}(\delta_{ik}b_{jp}b_{pl}+\delta_{il}b_{jp}b_{pk}+b_{ip}b_{pk}\delta_{jl}+b_{ip}b_{pl}\delta_{jk}) \end{array}$$

#### 2.3.2. Notes on Developing Subroutines 

In this study, a subroutine program based on Equation (33) was used to verify the superiority of the Yeoh_revised model (incompressible) in predicting SAC by FEM, and it was compared with the Yeoh and Ogden models. Since the Jacobi matrix expressed in Equation (33) is derived based on the premise of incompressibility, UMAT or UHYPER subprograms can be directly developed [47]. 

#### 2.3.3. Verification of the Equivalent CAE Simulation Design for the ET (SAC) Test 

**Figure 5:**
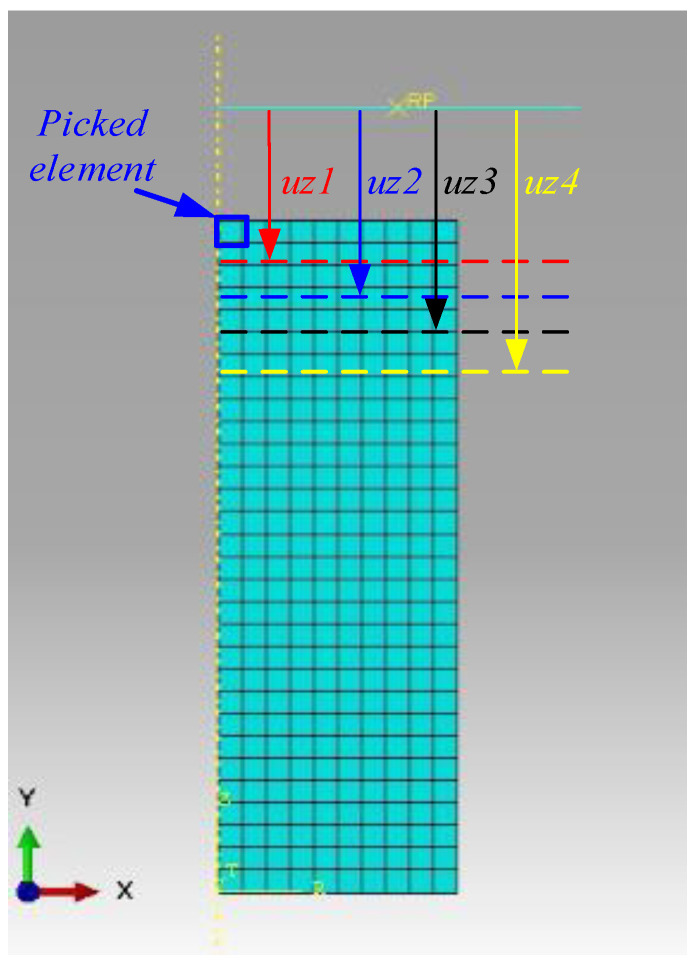
The FEM schematic diagram for rubber SAC.

On the premise that HNBR material volume is incompressible, combined with experimental data obtained at constant temperature (such as 120 °C), different λ_i_ values under ET boundary conditions were converted to different compressive axial displacements (uzi) based on Equation (1) (see Table 1). The contact stress between the picked mesh element and pressure plate was extracted, as shown in Figure 5, by using an axisymmetric model for calculations; the finite element mesh type of rubber material is CAX4H, and the number of CAX4H is 300. The pressure plate is designated as an analytical rigid body, its mesh type is RAX2, and the pressure plate and rubber are set to be in frictionless contact (equivalent to ET). The contact stresses under different λ_i_ are plotted together in Figure 8, which is based on the three constitutive models obtained using Equations (13), (16) and (19), respectively. In addition, the Yeoh_revised Jacobi matrix was derived on the basis of Equation (33), and the other two Jacobi matrix models usually exist in popular FEM software. 

## 3. Results and Discussion

### 3.1. Fitting Results Analysis

**Figure 6:**
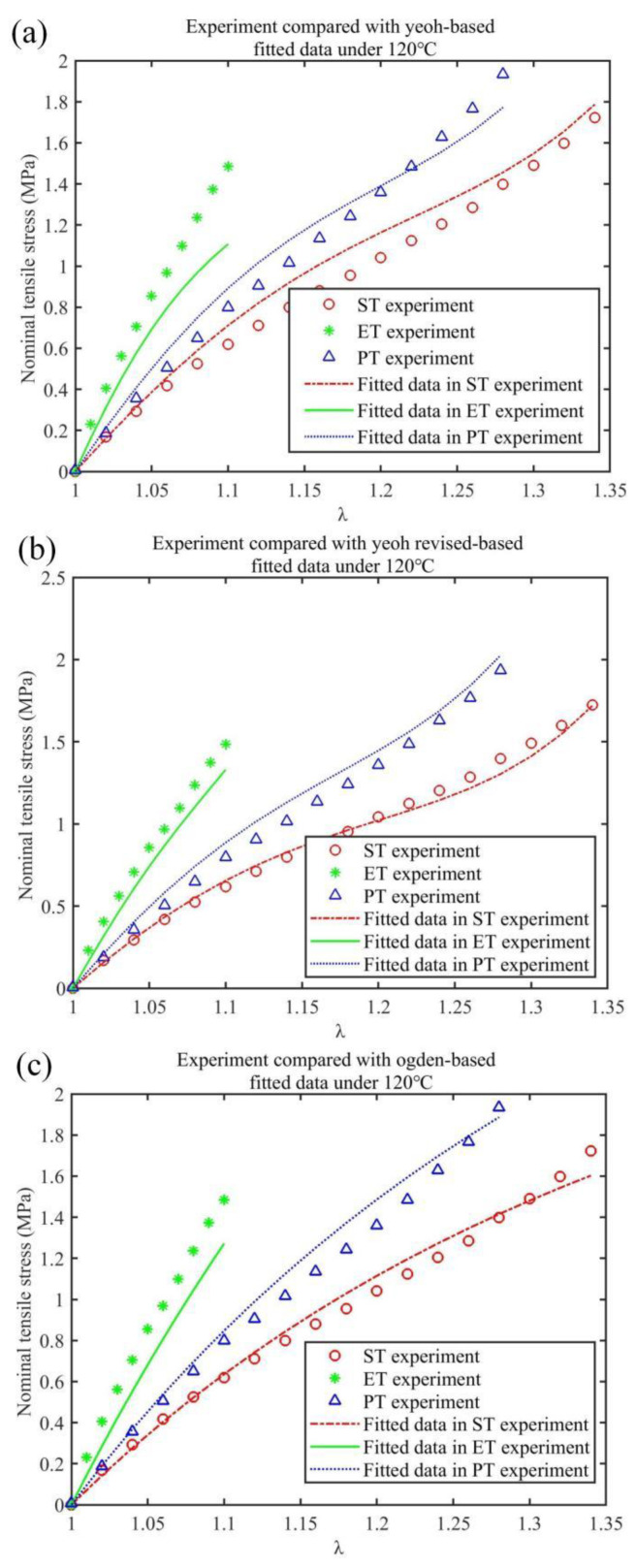
The results of three models fitted to HNBR stretching experiments at 120 °C: Yeoh model (**a**), Yeoh_revised model (**b**) and Ogden model (**c**).

**Figure 7:**
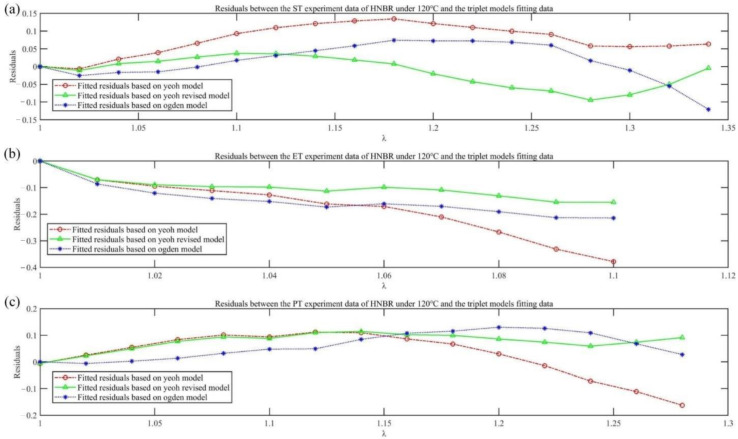
Residual analysis of three models for fitting results of HNBR tensile experiment at 120 °C: UT tensile (**a**), ET tensile (**b**) and PT tensile (**c**) results.

In Figure 6 and Figure 7, based on the three types (UT, ET and PT) of tensile experimental data of HNBR at 120 °C, the Yeoh_revised (N = 3) constitutive model’s overall goodness of fit (R^2^ = 0.9771) is the most accurate compared with the Yeoh (N = 3) constitutive and Ogden (N = 3) models, and the maximum negative fitting residuals are: −0.3782, −0.155, −0.2141, respectively; the Yeoh_revised model is improved compared to the Yeoh constitutive model, which predicts ET (or SAC) stress with the softness phenomenon. This is because the ET experimental fitting formula holds one more item $2C_{01}(\lambda^{3}-\lambda^{-3})$, which gradually increases as λ increases, than the Yeoh model in Equation (16). The obtained curve in Equation (16) shows a more obvious “upturning” phenomenon than that in Equation (13), which indicates better agreement with ET experimental data. Compared with the Ogden (N = 3) model, the Yeoh_revised (N = 3) model achieves a more accurate fit with fewer parameters (i.e., four), so it proves to be the most suitable for stress prediction in SAC deformation. Meanwhile, the Yeoh_revised (N = 3) model still has the smallest fluctuation in UT and PT tensile values, as shown in Figure 7b,c. Thus, in general, the Yeoh_revised (N = 3) model possesses the best fitting effect and merges the advantages of the other two fitting models. The fitting coefficient values of the three models, the deviation square sum (SSdev) and the goodness of fit (R^2^) are shown in Table 2. 

### 3.2. Equivalent FEM Verification Results

**Figure 8:**
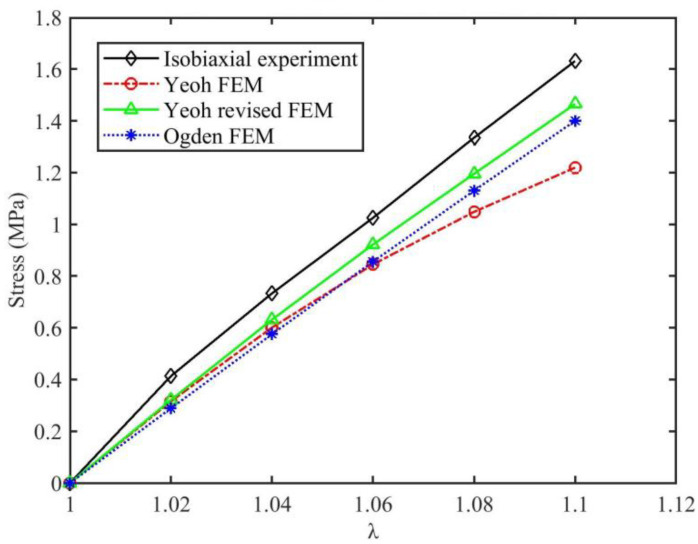
The SAC stress compared with FEM calculations at 120 °C.

In Figure 8, when the tensile ratio (λ) increases from 1 to 1.10 at an experimental temperature of 120 °C, the experimental values are consistently higher than the CAE-calculated values of the Yeoh_revised, Yeoh and Ogden models. Nevertheless, the three constitutive models produce results with different size degrees; in general, Yeoh_revised is the smallest, Yeoh is the largest, and Ogden is in the middle. For example, when λ is 1.10, the Yeoh_revised CAE calculation model is 10.17% smaller, while Yeoh_revised and Ogden CAE calculation models are 10.17% and 14.16% smaller, respectively, which again indicates that the Yeoh_revised model will significantly improve the soft SAC stress based on the Yeoh model. For other stretch ratios, the comparison between the CAE-calculated values of the three constitutive models and experiments is shown in Table 3.

Meanwhile, in Figure 8, it is clear that as λ increases from 1 to 1.10, the numerical values from the experiment and the CAE-calculated values of Yeoh_revised and Ogden are all linearly distributed, while the Yeoh CAE-calculated values are nonlinearly distributed, and their deviation from experimental values rises as λ increases. Therefore, the Yeoh model is seldom suitable for predicting SAC stress under large deformation.

In order to compare the difference between the experimentally fitted values (i.e., Equations (13), (16) and (19)) and the CAE-calculated values (i.e., Equation (33)) for the same λ, the fitted values of experimental SAC (ET) data were collated together with the CAE-calculated values of the three constitutive models in Table 4. The results demonstrate that the maximum deviation between the fitted values of each constitutive model and the corresponding CAE-calculated values never exceeds ±0.5% at 120 °C, which, importantly, meets the requirement for engineering accuracy, so the accuracy of the fitting parameters of each constitutive model obtained by the least squares method in this paper is proved. 

### 3.3. FEM Results of FRR under Static Compression

**Figure 9:**
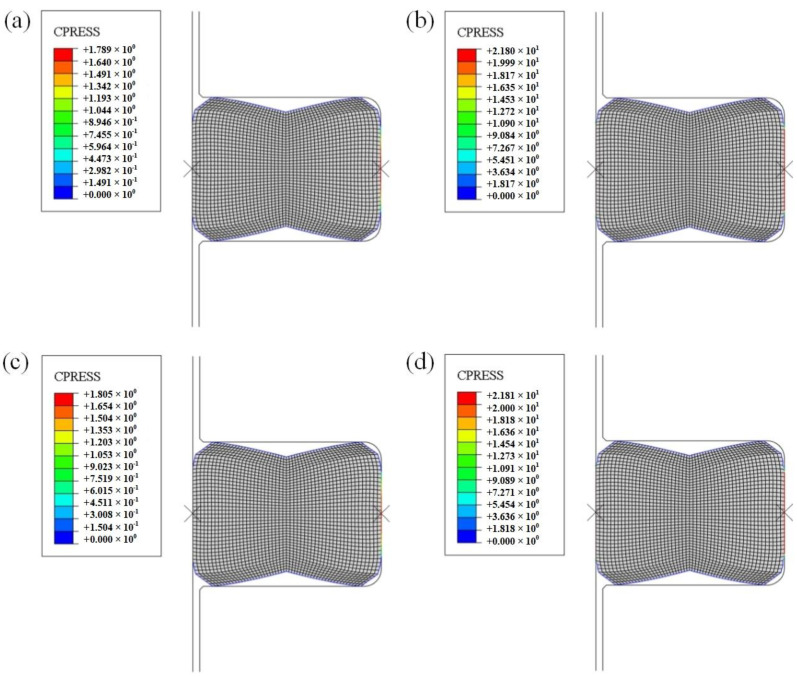
Contact stress cloud diagram of FRR in a roller bit: (**a**) pre-compression contact stress based on Yeoh model; (**b**) contact stress after fluid extrusion based on Yeoh model; (**c**) pre-compression contact stress based on Yeoh_revised model; (**d**) contact stress after fluid extrusion based on Yeoh_revised model.

**Figure 10:**
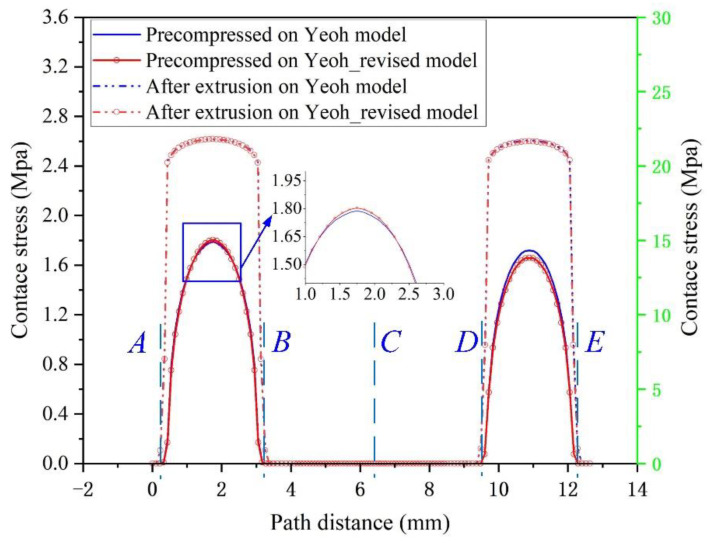
Contact stress on the contact path of FRR in a roller bit. In the contact path (green dotted line in Figure 1b), the critical point on the rotation surface contact with the outer and inner fluid zone is A and B, point C is the middle point in the contact path and the critical point on the outside surface contact with the outer and inner fluid zone is D and E.

The cloud diagram of contact stress FEM calculation results under FRR static compression is shown in Figure 9. For a more intuitive comparison, the contact stresses calculated based on two constitutive models in the contact path (Figure 1b) is extracted and plotted in Figure 10, where the static pre-compression contact stresses are basically the same distribution, and the difference between the contact stresses after being squeezed by fluid is also subtle. In comparison, based on the Yeoh_revised model, the contact stress value on the rotation surface is slightly larger than the value calculated by the Yeoh model between points A and B in the contact path, while the contact stress value on the outside surface between points D and E shows the opposite pattern. After being squeezed by fluid, the contact stress of the two contact surfaces increases above the fluid pressure (i.e., 20 MPa) in the cavity. Meanwhile, the contact stress after fluid extrusion based on the two constitutive models is basically the same, which indicates that the FRR structure with this structural parameter set would ensure that it is sealed. The Mises stress expression is shown in Equation (34).

(34) $$\sigma =\sqrt{\frac{1}{2}[{\left(\sigma_{1}-\sigma_{2}\right)}^{2}+{\left(\sigma_{2}-\sigma_{3}\right)}^{2}+{\left(\sigma_{3}-\sigma_{1}\right)}^{2}]}$$

**Figure 11:**
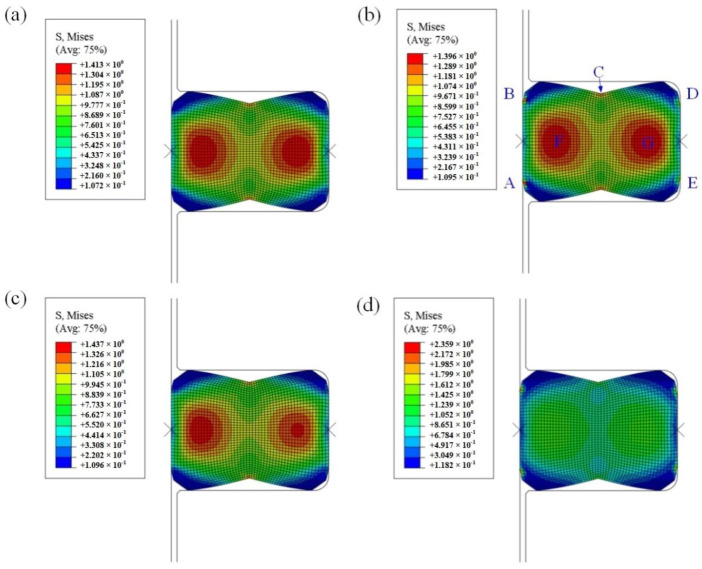
Mises stress cloud diagram of FRR in a roller bit: (**a**) pre-compression Mises stress based on Yeoh model; (**b**) Mises stress after fluid extrusion based on Yeoh model; (**c**) pre-compression Mises stress based on Yeoh_revised model; (**d**) Mises stress after fluid extrusion based on Yeoh_revised model. In the contact path (green dotted line in Figure 1b), the critical point on the rotation surface contact with the outer and inner fluid zone is A and B, point C is the middle point in the contact path and the critical point on the outside surface contact with the outer and inner fluid zone is D and E.

Under static compression, the maximum Mises stress calculated based on the two constitutive models is located in the F, G and C areas of the FRR section, as shown in Figure 11a,c, and only the maximum Mises stress values are different. According to Equations (20a) and (34), the Yeoh_revised model predicts principal stress σ_i_ (i = 1, 2, 3) values more accurately in each direction, so it also desired the value of Mises stress more precise. For instance, the maximum Mises stress value obtained based on the Yeoh_revised model is 1.437 MPa, which is greater than the Yeoh model value of 1.413 MPa. 

**Figure 12:**
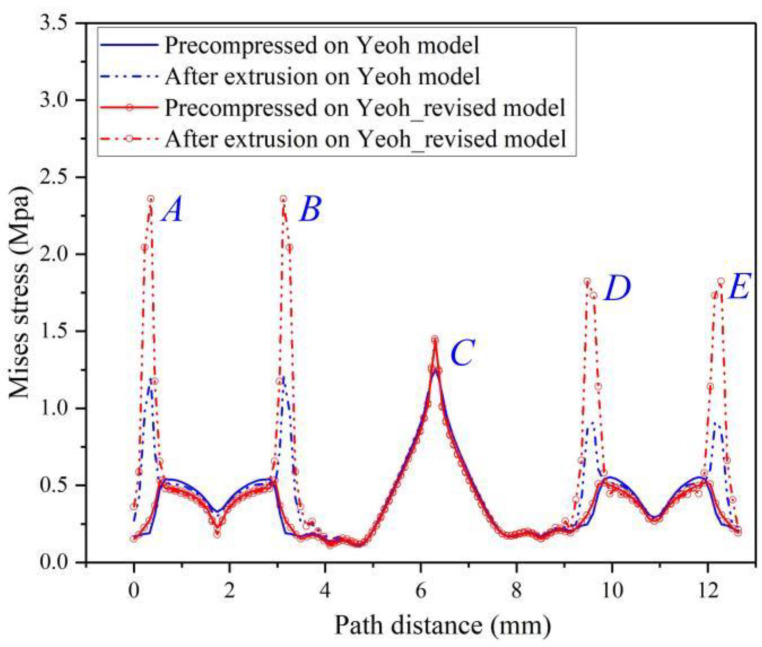
Mises stress on the contact path of FRR in roller bit. In the contact path (green dotted line in Figure 1b), the critical point on the rotation surface contact with the outer and inner fluid zone is A and B, point C is the middle point in the contact path and the critical point on the outside surface contact with the outer and inner fluid zone is D and E.

After being squeezed by fluid, the maximum Mises stress also occurs in areas where the two sealing surfaces are in contact with the fluid, i.e., A, B, D and E. It is also found that the cloud graph in Figure 11d is not consistent with Figure 11b; based on the Yeoh_revised model, the maximum Mises stress after fluid extrusion is mainly distributed in the contact areas between the two sealing contact surfaces and the liquid cavity (i.e., A, B, D and E), while the Yeoh model hardly reflects this distribution law. Extracting the Mises stress values on the contact path (Figure 1b), as shown in Figure 12, indicates that Mises stress values at the contact areas (i.e., A, B) are basically the same. Owing to the symmetrical deformation of the FRR, the contact areas are at the junction of the rotation sealing surface and fluid of both ends, and the two contact areas (D, E) on the outer sealing surface are also identical. Meanwhile, based on the two constitutive models, Mises stress concentration occurs in the four regions (A, B, D and E) after being squeezed by fluid (i.e., the FRR sealing process), and the Mises stress obtained by the Yeoh_revised model is twice the value obtained by the Yeoh model; the larger the value, the easier it is to age and harden in the four regions. Simultaneously, Mises stress in the contact areas (i.e., A and B) at the junction of the rotating surface and fluid is the maximum on the contact path, which indicates that this area is the most fragile part of the entire FRR structure and is likely to cause the sealing effect of the FRR to deteriorate [48]. 

## 4. FRR Field Application

**Figure 13:**
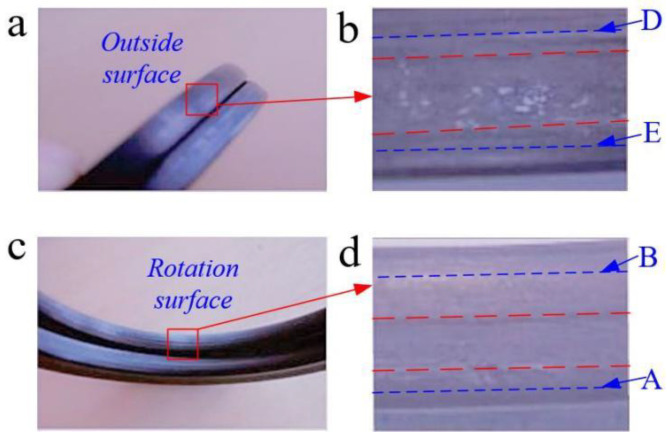
The on-site wear effect on FRR in a roller bit. The wear effect on the outside and rotation surfaces of FRR before and after use is shown in (**a**) and (**c**), respectively. The magnification of the outside and rotation surfaces’ wear effect is shown in (**b**) and (**d**), respectively. In the contact path (green dotted line in Figure 1b), the critical point on the rotation surface contact with the outer and inner fluid zone is A and B, the critical point on the outside surface contact with the outer and inner fluid zone is D and E.

Taking the well named MaHW6415 as an example, the lithology is gray mudstone, the estimated ambient temperature is 90 °C, and the pump pressure is 20 MPa (fluid pressure difference: ±0.5 MPa). After the total number of rotations of the roller bit reached about 980,000 cycles, the FRR was extracted, its appearance was checked, and the wear condition was analyzed, as shown in Figure 13. 

**Figure 14:**
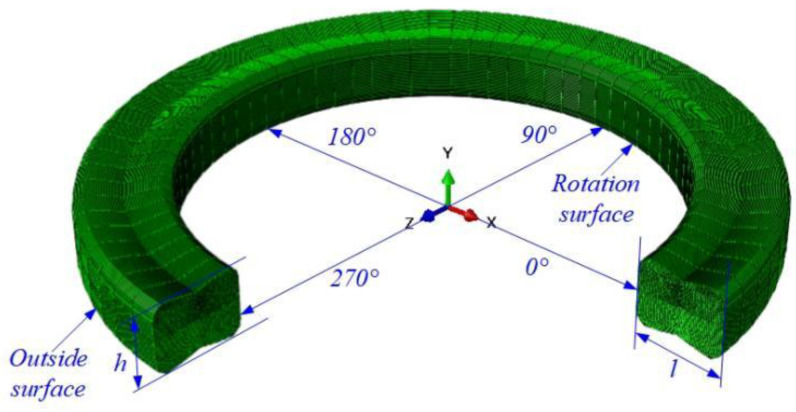
Schematic diagram of wear measurement of FRR in a roller bit.

There are worn lines on the rotation surface and outside surface in Figure 13b,d. The larger the distance between the center line of the stress concentration area (i.e., thick dotted line) and the wear gradient boundary line (i.e., thin dotted line), the larger the hardened area of the rubber contact surface, the more severe the aging of the rubber contact surface, and the worse the sealing effect. Meanwhile, the distance between the thick dotted line and the thin dotted line of the cross-sectional stress concentration area in Figure 13d is higher than that in Figure 13b, which demonstrates that the maximum Mises stress on the rotation sealing surface is higher than that on the outside sealing surface. Consequently, the spacing distance will be reduced by optimizing the FRR structural parameters to reduce the maximum Mises stress value. The schematic diagrams and test results of rotation and outside surfaces are shown in Figure 14 and Table 5, respectively. 

In Table 5, the inner-diameter wear value of the rotation surface before and after use is 0.52 mm; the maximum wear amount of the section length is about 0.30 mm at 90° or 180°, respectively, so eccentric wear occurs, which shows that the FRR is not completely antisymmetrically deformed inside the roller drill. The hardness of the FRR rotation surface is hardened by 11HA before and after use, while the outside surface is only hardened by 4HA, which also proves that the maximum Mises stress on the rotation sealing surface is higher than that on the outside sealing surface in actual operation. 

## 5. Conclusions 

This paper mainly focuses on the SAC contact stress and Mises stress of the Yeoh_revised model compared to the Yeoh model, which was also applied to evaluate the sealing effect of an FRR in a roller bit under SAC deformation. The results clearly show that the Yeoh_revised model can predict the Mises stress more accurately than the Yeoh model and provides theoretical guidance for mitigating the aging of FRRs and ensuring a more stable seal. It also further provides specifications for its size optimization. The following important conclusions are drawn from this research:

1. Comparing the fitted values with the FEM-calculated data of three constitutive models, it is demonstrated that the maximum deviation between the fitted value of each constitutive model and the corresponding CAE-calculated value never exceeds ±0.5% at 120 °C, which proves the accuracy of the fitting values of the parameters of each constitutive model obtained through the least squares method in this paper.
2. Compared with the Yeoh model, the Yeoh_revised and Ogden models both address the soft phenomenon encountered by the Yeoh constitutive model when predicting stress in ET (SAC) tensile tests. Moreover, the Yeoh_revised model shows the greatest improvement, with an R^2^ up to 0.9771, in fitting the experimental values, and its maximum underestimation is reduced to half of that of the Yeoh model.
3. The Yeoh_revised constitutive model is the most accurate in fitting the experimental data. Compared with Ogden, it achieves more accurate fitting with fewer parameters (i.e., four), while the number of fitted parameters (i.e., six) is higher in the Ogden model; therefore, it is more suitable for Mises stress analysis of an FRR in a roller bit under SAC deformation.
4. On the premise of ensuring the stability of sealing FRR contact stress, the maximum Mises stress obtained with the Yeoh_revised model is 1.437 MPa greater than the Yeoh model’s value of 1.413 MPa before FRR extrusion by fluid. The Yeoh_revised model is more accurate in predicting Mises stress. In the sealing process (i.e., after FRR extrusion by fluid), the Mises stress obtained with the Yeoh_revised model is twice the value obtained with the Yeoh model, which provides a more reasonable prediction for reducing FRR aging and further ensuring a more stable seal. It also provides specifications for its size optimization in the future.

## Figures and Tables

**Table 1:** SAC displacements corresponding to different tensile ratios λ_i_.

λ_i_	1	1.02	1.04	1.06	1.08	1.10
uzi	0	6.16 mm	7.26 mm	8.30 mm	9.28 mm	10.21 mm

**Table 2:** Comparison of the fitted results of tensile stress based on three models.

Yeoh		Yeoh_Revised		Ogden	
*T*	120 °C	*T*	120 °C	*T*	120 °C
*C*_10_	1.3745	*C*_10_	0.36	*u*_1_	0.8152
*C*_20_	−1.4273	*C*_20_	−1.3323	*α*_1_	−0.0003
*C*_30_	2.3639	*C*_30_	3.0578	*u*_2_	0.8152
/	/	*C*_01_	0.9965	*α*_2_	0.0005
/	/	/	/	*u*_3_	0.8152
/	/	/	/	*α*_3_	−0.0002
*SS_dev_*	0.7124	*SS_dev_*	0.2659	*SS_dev_*	0.6465
*R* ^2^	0.9385	*R* ^2^	0.9771	*R* ^2^	0.9442

**Table 3:** Comparison between CAE-calculated and experimental values.

	λ	Test_Data	Yeoh_Cae	Deviation	Yeoh_Re_Cae	Deviation	Ogden_Cae	Deviation
T = 120 °C	1.00	0.0000	0.0000	0	0.0000	0	0.0000	0
	1.02	0.4137	0.3159	−23.65%	0.3213	−22.34%	0.2892	−30.10%
	1.04	0.7333	0.601	−18.04%	0.6315	−13.88%	0.5753	−21.55%
	1.06	1.0250	0.8451	−17.55%	0.9219	−10.06%	0.8559	−16.50%
	1.08	1.3346	1.048	−21.48%	1.195	−10.46%	1.1300	−15.33%
	1.10	1.6309	1.219	−25.26%	1.465	−10.17%	1.4000	−14.16%

Notes: deviation = (xx_cae − test_date)/test_date × 100%.

**Table 4:** Comparison between CAE-calculated and fitted values.

T = 120 °C							
λ	Yeoh_Cae	Yeoh_Fit	Deviation	λ	Ogden_Cae	Ogden_Fit	Deviation
1.00	0.0000	0.0000	0	1.00	0.0000	0.0000	0
1.02	0.3159	0.3173	−0.43%	1.02	0.2892	0.2905	−0.46%
1.04	0.6010	0.6008	0.04%	1.04	0.5753	0.5752	0.01%
1.06	0.8451	0.8438	0.15%	1.06	0.8559	0.8546	0.15%
1.08	1.0480	1.0466	0.13%	1.08	1.1300	1.1290	0.09%
1.10	1.2190	1.2157	0.27%	1.10	1.4000	1.3959	0.29%
λ	Yeoh_re_cae	Yeoh_re_fit	Deviation				
1.00	0.0000	0.0000	0				
1.02	0.3213	0.3227	−0.43%				
1.04	0.6315	0.6315	0.01%				
1.06	0.9219	0.9207	0.13%				
1.08	1.1950	1.1934	0.13%				
1.10	1.4650	1.4608	0.29%				

Notes: deviation = (xx_cae − xx_fit)/xx_cae × 100%.

**Table 5:** Flat sealing ring size and hardness measurements before and after use.

Object	Bore Size/mm	Length of Different Phase-Angle Cross-Sections (l)				Height of Different Phase-Angle Cross-Sections (h)				Hardness/HA	
		0°	90°	180°	270°	0°	90°	180°	270°	Outside Surface	Rotation Surface
Before use	54.70	6.25	6.25	6.25	6.25	3.90	3.90	3.90	3.90	90	90
After use	55.22	6.03	5.96	5.95	6.05	3.92	3.92	3.88	3.89	94	101

## Data Availability

Not applicable.

## Appendix A

The derivation process of Equation (27) is as follows:

(A1) $$II_{ijkl}^{sym}=0.5(II_{ijkl}+{\bar{II}}_{ijkl})=0.5(\delta_{ik}\delta_{jl}+\delta_{il}\delta_{jk})$$

where $II=I\bar{⊗}I,\bar{II}=I\underline{⊗}I$ is a minor symmetric fourth-order unit tensor [38].

According to the symmetry of D:

(A2) $$\begin{array}{l} D_{ip}=II_{ipkl}^{sym}D_{kl}=0.5(\delta_{ik}\delta_{pl}+\delta_{il}\delta_{pk})D_{kl}\\ D_{pj}=II_{pjkl}^{sym}D_{kl}=0.5(\delta_{pk}\delta_{jl}+\delta_{pl}\delta_{jk})D_{kl} \end{array}$$

Substitute in:

(A3) $$\begin{array}{l} D_{ip}{\tilde{b}}_{pj}=0.5(\delta_{ik}{\tilde{b}}_{jl}+\delta_{il}{\tilde{b}}_{jk})D_{kl}\\ {\tilde{b}}_{ip}D_{pj}=0.5({\tilde{b}}_{ik}\delta_{jl}+{\tilde{b}}_{il}\delta_{jk})D_{kl} \end{array}$$

(A4) $$\begin{array}{l} D_{ip}I_{pj}=0.5(\delta_{ik}\delta_{jl}+\delta_{il}\delta_{jk})D_{kl}\\ I_{ip}D_{pj}=0.5(\delta_{ik}\delta_{jl}+\delta_{il}\delta_{jk})D_{kl} \end{array}$$

(A5) $$\begin{array}{l} D_{ip}{\tilde{b}}_{pq}{\tilde{b}}_{qj}=0.5(\delta_{ik}{\tilde{b}}_{jp}{\tilde{b}}_{pl}+\delta_{il}{\tilde{b}}_{jp}{\tilde{b}}_{pk})D_{kl}\\ {\tilde{b}}_{ip}{\tilde{b}}_{pq}D_{qj}=0.5({\tilde{b}}_{ip}{\tilde{b}}_{pk}\delta_{jl}+{\tilde{b}}_{ip}{\tilde{b}}_{pl}\delta_{jk})D_{kl} \end{array}$$

According to Equation (21b), Equations (A6) and (A7) can be obtained:

(A6) $$\tau =J\sigma =F\cdot S\cdot F^{T}=2C_{10}J^{-\frac{2}{3}}[b-\frac{trb}{3}I]+\frac{2}{D_{1}}J(J-1)I$$

(A7) $$\begin{array}{l} 2H:D=2C_{10}(D\cdot \tilde{b}+\tilde{b}\cdot D)\\ -[2C_{10}\frac{tr\tilde{b}}{3}-\frac{2}{D_{1}}(J^{2}-J)](D\cdot I+I\cdot D) \end{array}$$

Substituting Equations (A3) and (A4) into Equation (A7), Equation (27) can be obtained.

## Appendix B

Equation (32) can be redefined as Equation (A8):

(A8) $$\begin{array}{l} S=\tilde{S}+S^{vol}=\sum_{i=1}^{3}2iC_{i0}(I_{C}-3)i-1I+2C_{01}(I_{C}I-C)+J\gamma C^{-1}\\ =S_{C_{10}}+S_{C_{20}}+S_{C_{30}}+S_{C_{01}}+S^{vol} \end{array}$$

where:

(A9) $$S_{C_{10}}=2C_{10}I$$

(A10) $$S_{C_{20}}=4C_{20}(I_{C}-3)I$$

(A11) $$S_{C_{30}}=6C_{30}{\left(I_{C}-3\right)}^{2}I$$

(A12) $$S_{C_{01}}=2C_{01}(I_{C}I-C)$$

(A13) $$S_{vol}=J\gamma C^{-1}$$

According to the definition in Equation (25), Equation (A14) is obtained from Equation (A9):

(A14) $$C_{C_{10}}^{\tan}=2\frac{\partial S_{C_{10}}}{\partial C}=0$$

(A15) $$C_{C_{20}}^{\tan}=2\frac{\partial S_{C_{20}}}{\partial C}=8C_{20}I⊗I$$

(A16) $$C_{C_{30}}^{\tan}=2\frac{\partial S_{C_{30}}}{\partial C}=(24C_{30}I_{C}-72C_{30})I⊗I$$

(A17) $$C_{C_{01}}^{\tan}=2\frac{\partial S_{C_{01}}}{\partial C}=4C_{01}I⊗I-4C_{01}I\bar{⊗}I$$

(A18) $$C_{vol}^{\tan}=2\frac{\partial S_{vol}}{\partial C}=\gamma JC^{-1}⊗C^{-1}-\gamma J(C^{-1}\bar{⊗}C^{-1}+C^{-1}\underline{⊗}C^{-1})$$

Combining Equations (A14)–(A18), Equation (A19) is obtained:

(A19) $$\begin{array}{l} C^{\tan}=(8C_{20}+24C_{30}I_{C}-72C_{30}+4C_{01})I⊗I-4C_{01}I\bar{⊗}I\\ +\gamma JC^{-1}⊗C^{-1}-\gamma J(C^{-1}\bar{⊗}C^{-1}+C^{-1}\underline{⊗}C^{-1}) \end{array}$$

According to Equation (21a), Equation (A19) becomes Equation (A20), which is the spatial tangent elasticity tensor (***L***) of the Yeoh_revised model (incompressible).

(A20) $$\begin{array}{l} L=(8C_{20}+24C_{30}I_{b}-72C_{30}+4C_{01})b⊗b-4C_{01}b\bar{⊗}b\\ +\gamma JI⊗I-\gamma J(I\bar{⊗}I+I\underline{⊗}I) \end{array}$$

Substituting Equations (A3)–(A5) into Equation (A20), the fractional formula of **L** of the Yeoh_revised model (incompressible) can be obtained, as shown in Equation (A21).

(A21) $$\begin{array}{l} L_{ijkl}=(8C_{20}+24C_{30}I_{b}-72C_{30}+4C_{01})b_{ij}b_{kl}-4C_{01}b_{ik}b_{jl}\\ +\gamma J\delta_{ij}\delta_{kl}-\gamma J(\delta_{ik}\delta_{jl}+\delta_{il}\delta_{jk}) \end{array}$$

Similarly, on the basis of Equation (32), according to Equation (21b), Equations (A22) and (A23) can be obtained.

(A22) $$\begin{array}{l} \tau =J\sigma =F\cdot S\cdot F^{T}=\left[\sum_{i=1}^{3}2iC_{i0}{\left(I_{b}-3\right)}^{i-1}+2C_{01}I_{b}\right]b\\ -2C_{01}b\cdot b+J\gamma I \end{array}$$

(A23) $$\begin{array}{l} 2H:D=\left[\sum_{i=1}^{3}2iC_{i0}{\left(I_{b}-3\right)}^{i-1}+2C_{01}I_{b}\right](D\cdot b+b\cdot D)\\ -2C_{01}(D\cdot b\cdot b+b\cdot b\cdot D)+J\gamma (D\cdot I+I\cdot D) \end{array}$$

Substituting Equations (A3)–(A5) into Equation (A23), Equation (A24) can be obtained.

(A24) $$\begin{array}{l} 2H_{ijkl}=\left[\sum_{i=1}^{3}iC_{i0}{\left(I_{b}-3\right)}^{i-1}+C_{01}I_{b}\right](\delta_{ik}b_{jl}+\delta_{il}b_{jk}+b_{ik}\delta_{jl}+b_{il}\delta_{jk})\\ -C_{01}(\delta_{ik}b_{jp}b_{pl}+\delta_{il}b_{jp}b_{pk}+b_{ip}b_{pk}\delta_{jl}+b_{ip}b_{pl}\delta_{jk})+J\gamma (\delta_{ik}\delta_{jl}+\delta_{il}\delta_{jk}) \end{array}$$

Combining Equations (A21) and (A24), the fractional formula of $\hat{L}$ of the Yeoh_revised model (incompressible) can be obtained, as shown in Equation (A25).

(A25) $$\begin{array}{l} {\hat{L}}_{ijkl}=L_{ijkl}+2H_{ijkl}=(8C_{20}+24C_{30}I_{b}-72C_{30}+4C_{01})b_{ij}b_{kl}\\ -C_{01}(\delta_{ik}b_{jp}b_{pl}+\delta_{il}b_{jp}b_{pk}+b_{ip}b_{pk}\delta_{jl}+b_{ip}b_{pl}\delta_{jk})-4C_{01}b_{ik}b_{jl}+\gamma J\delta_{ij}\delta_{kl}\\ +\left[\sum_{i=1}^{3}iC_{i0}{\left(I_{b}-3\right)}^{i-1}+C_{01}I_{b}\right](\delta_{ik}b_{jl}+\delta_{il}b_{jk}+b_{ik}\delta_{jl}+b_{il}\delta_{jk}) \end{array}$$

Finally, on the basis of Equation (A25), the Yeoh_revised model (incompressible) Jacobi matrix component ***C_ijkl_*** is obtained, as shown in Equation (33).
